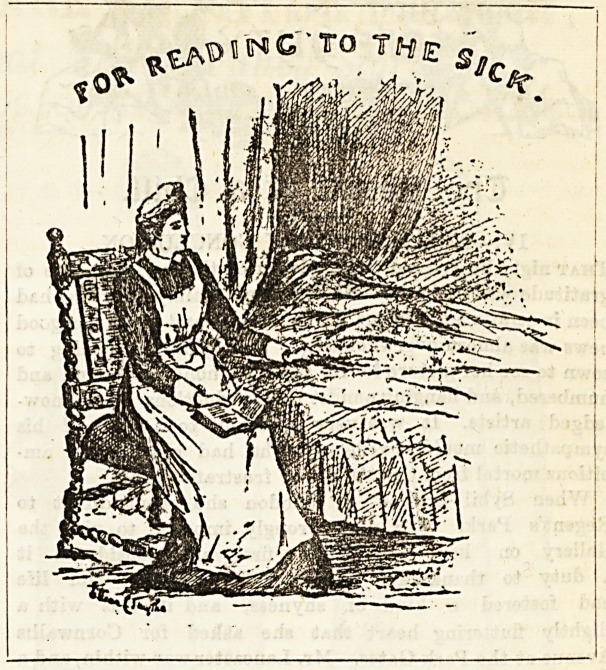# The Hospital Nursing Supplement

**Published:** 1892-10-29

**Authors:** 


### "The Hospital" Nursing Mirror.

Being the Extra Nursing Supplement of "The Hospital" Newspaper.

[Contributions for this Supplement should be addressed to the Editor, The Hospital, 140, Strand, London, W.C., and should have the word "Nursing" plainly written in left-hand top corner of the envelope.]

### En Passant.

LINCOLN DIOCESAN CONFERENCE. — District nursing of the sick poor has been one of the main subjects of discussion at this Conference, and it has been suggested that a Special Committee should be formed to inquire into the best system now in use in the various parts of England. The Ockley system, originated by Miss Broadwood, and the Q.V.J.I.N. Rural District Branch, were all described by the Rev. J. S. Warren, and the resolution was ultimately carried that both systems should be considered, and the matter brought before the county at large. The scattered villages of Lincolnshire will require a very clear-headed committee to work the scheme both economically and well.

ST. LUKE'S HOME, VANCOUVER.—A September cutting from the Vancouver *Weekly World* has been sent us by a friend of Sister Frances, whom our Hospital readers know very well as working hard in Vancouver to keep open two homes where trained nursing can be had by rich or poor. The cutting describes a grand bazaar and garden party at Chilliwack, which was held for the benefit of the Home there, and which was mainly arranged and carried to complete success by the energy of Mrs. and Miss Corbett. At the end of the bazaar, Mr. Allen, in speaking of the good work Sister Frances and her colleagues are doing, assured all present that poverty or distress would never be sent away from the home unrelieved. A sum of a hundred and twenty-five dollars was realised. Our readers will remember an appeal from Sister Frances which appeared on October 8th. Anyone who could spare her a trifle would earn her deep gratitude, and the slightest help will be acknowledged if sent to the Rev. H. Fiennes Clinton, Cromwell Rectory, Newark, England.

FOUNDLINGS AND FEVERS.—In our issue of May 7th we inserted a paragraph headed "Facts and Foundlings," calling attention to the extraordinary arrangement which continued to admit swarms of Sunday visitors to an institution when there were nearly a dozen cases of scarlatina on the premises. The infected condition of the place at that time can hardly be doubted in the face of the Secretary's letter to the *Times* of Oct. 14, in which he writes: "Since the end of March last nearly 200 children have had a mild form of scarlet fever." There are only 360 children at the Foundling when it is quite full, and, therefore, this percentage is amazingly high, especially as we must remember that they are not youngsters attending an ordinary school where day pupils might be credited with the conveyance of infection. Here the children are always, to a certain extent, isolated, and, therefore, the root of the evil is within their own domain. Dr. Sykes' letter in the *Times* of the 25th inst. draws attention to the unaccountable absence of any special building for isolation purposes within the grounds of the Foundling Hospital, although the site proposed for this was staked out in the early spring. He says, "Infection being once introduced, the means of properly isolating cases in a separate building should exist in this institution, as in every well-regulated residential school." The typhoid epidemic last year laid low many adults, whereas the scarlet fever this year has confined its attacks to the poor little children. Perhaps some of the liberal public who flock to the Sunday show will exhibit sufficient interest in the sanitary condition of the "charity" they support to demand a full and exhaustive inquiry into the "facts" we have mentioned.

AMERICAN JOTTINGS.—The last number of the "Trained Nurse" is an interesting one. Dr. Potter's account of the R.N.P.F. still continues, and, by the bye, a "Boston Nurse" writes a capital letter criticising the sentimental people who have condemned the fund as "charity." An address by Mrs. Walker, North-Western Hospital, Minneapolis, on the "Progressive and Unprogressive Nurse," is well worth reading. The progressive nurse, we are told, will "bring to her work that gospel of faithfulness in the 'least' things which is the keynote to all progress and perfection." Miss Isabel Hampton, of the Johns Hopkins Hospital is working on the Maryland Committee on Women's Exhibits at the World's Fair. Maryland nurses' work will find a prominent place in this section. Miss Louise Parsons, who received the Royal Red Cross in the Egyptian campaign, has been appointed Superintendent of Nurses to the City and County Hospital, St. Paul, Minn. Miss Elizabeth Penny, who trained at University College Hospital, has been appointed District Nurse at the Memorial Hospital.

JEDBURGH DISTRICT NURSING ASSOCIATION.—The report of a first year's work in nursing at Jedburgh should be an encouragement to others about to start local branches. The association owes not only its existence, but in a great measure its success, to the Marchioness of Lothian's continuous efforts. She has generally presided over the monthly meeting of a business-like committee, and she drew up the original rules on which they should act. Nurse Rae commenced work on September 3rd, and she has earned a great deal of gratitude during an exceedingly heavy year. Typhoid fever sent her a great deal of work, but sanitary precautions will probably keep that off her list of cases this year. The Jedburgh Nursing Association, like many others, has started a collection of invalid appliances, a water bed has been given to the committee, and many other things, without the help of which the nurses' work becomes very much more difficult. We take this opportunity of reminding our readers that there is nobody to beat a district nurse for finding a use for everything, and that we are always willing and able to help those who have any invalid appliances to give away, to find a place where their gifts will be almost invaluable.

WHERE TO GO.—It is not always easy in the winter afternoons and evenings to think of how to spend an hour or two off duty, yet it ought to be a rule of every nurse's life to take her rest and recreation as thoroughly as it would be to perform any other duty. And it is a good thing to remember that one must never let one's work in life narrow down one's experience or sympathy with other phases of life. Some of us are apt to talk and think and dream of our profession too much as though it were the only one in life, and so we sometimes are accused of having no ideas outside our little lives. The great antidote to this is to imbibe new ideas from outside sources, and it is not a very difficult thing to do in these days when free lectures abound, and when a shilling will take us to many places. Nurses see so much of sorrow that it is good to go out and see some of the smiles of life, else how can they expect to impart the cheerfulness and hope which is one of the best medicines a sick man can be given. A concert, or an hour at a picture gallery, whichever way our tastes may lie, will prove tonics not to be sneered at, and we say this, remembering fully that nurses are often very weary and feel as though a chair and a novel is all the recreation they care to be bothered with; for all that, we maintain that it is not a feeling to give way to, and we hope our small signposts which occasionally appear in the "Mirror" are not put up altogether in vain.

### Lectures for Asylum Attendants.

By William Harding, M.B.

V.—FOOD (*continued*).

Acute melancholics will generally require great quantities of food. In many cases before their arrival at the asylum they have for days or weeks been insufficiently nourished. The friends are unable to exercise that amount of moral control which is wanted, and the patients are in some instances positively half-starved. Their feeding must be begun carefully and steadily persisted in. If the nurse can make her patient gain in weight she may reasonably expect mental improvement. It is amongst the melancholics that the most obstinate cases of refusal of food occur. Frequently they will be found to have some derangement of the digestive system, and the discomfort they feel after meals may be the origin of the common delusion that their food is poisoned. The administration of nourishment in proper amounts and at proper times is all important, but at the same time presents many difficulties. The nurse must strive to acquire some control over her patients, and this she must endeavour to obtain at the very beginning of her acquaintance with them. While she tries to gain their confidence and to make them feel that she is their real friend, let her not forget the old saying about the evil effects of familiarity. The admission of a patient to the asylum, and her entrance upon entirely new surroundings in which a regular routine must be followed, is an excellent opportunity to insist upon a return to normal habits as regards food as well as other matters. Often those who come with dreadful reputations from outside give no trouble whatever, but fall at once into the ordinary ways of the institution.

The worst examples of refusal of food occur in those whose delusions are of a religious character. These are often very obstinate and difficult to manage. Among the delusions which we may meet with every day are the following: God has commanded that no food will be taken: The food is poisonous or contains filth: The patient has neither throat nor stomach: The bowels are blocked, &c. Such cases as these may require forcible feeding.

There is another class who, from some hysterical notion of attracting attention to themselves, will begin to decline to take food. Forcible feeding or any fuss made over them is what they would prefer. They are not likely to injure their health by abstinence, and the best treatment is to let them severely alone. After a short fast they will return to their food with their digestive apparatus all the better for the rest it has had. There are other individuals who will obstinately refuse to eat so long as any notice is taken of them, but who will do so if they think they are unobserved, and will even pilfer food from other patients, and eat it on the sly. Not infrequently a single feeding with the tube is sufficient to induce the patient to give up her determination to starve herself; others will persist in their refusal for long periods, and in some instances appear to enjoy the operation of being fed. In this, as in most other respects, male lunatics are more easily managed than those of the opposite sex.

A nurse should never exercise any force in feeding a patient. If the patient will not take food without a struggle she should leave the matter for the doctor to decide whether forcible feeding is necessary in that particular instance, and by what method food is to be administered. It is at times a matter of great difficulty for the doctor to decide whether it is wise to use the tube, and in some cases of organic disease useless suffering may be entailed upon the patient by misguided zeal for her welfare. In cases where there is no weakness of the digestive system the stomach pump possesses one great advantage. By it ordinary and varied diet can be given in the form of meat and vegetables which have been crushed, passed through a fine sieve, and moistened with gravy or beef tea. The details of feeding can be best explained practically in the wards.

We know that even in the nursing of sane persons it is not always easy to induce the invalid to take sufficient food, but the difficulties are often greater when disease attacks the chronic lunatic; much depends upon the amount of influence the nurse has gained over her people. Occasionally we find that some inter-current disease has a good effect upon the mental condition of a patient who has been refusing food. This chance of eradicating bad habits and of teaching better ones would be utilized by a capable nurse. The attempt to keep the insane sick upon special diet, as for example, in diabetes, is generally futile.

Difficulties also arise with regard to the administration of medicines, and at times are so great that it is next to impossible to carry on any continuous medical treatment. Some lunatics will with pleasure swallow the filthiest concoctions and ask for more; others will demand purgatives daily. There is generally little difficulty in getting epileptics to take drugs, but it is often otherwise in the very cases to whom we are most anxious to give them. The hypodermic method, when available, is very useful. The questions that will often arise are whether it is better to omit medicines, give them forcibly, or to try to deceive the patient into taking them. The last method is not one to be recommended. It is sure to fail if any continuous administration is desired, and frequently is unnecessary for single doses. If the patient herself does not see the drug put into her food others may do so, and a spirit of suspicion is apt to arise. In cases where the patient is so insane as to be unaware of the nature of what she takes, there are no objections to the method, and at times it may be useful in giving single doses to chronic lunatics. When the question of nourishment is of the first importance, as it so often is, the plan should not be used. The insane person may have sufficient intelligence to detect the presence of the drug, and may begin to refuse her food. Those also who have delusions that their food is poisoned should certainly not have it tampered with in this way. The endeavour to gain the lunatic's confidence is not very successful when the patient discovers that you are the individual who has been placing noxious articles in her diet. It is better to produce the dose openly as medicine. As a rule a confident manner, with an assurance that it is for the patient's good and must be taken are sufficient when the administration is not long-continued. Here again it must be remembered that no force must be used by the nurse.

### Royal British Nurses' Association.

At a Council meeting of the R.B.N.A. on Friday evening, October 21st, Sir William Savory was in the chair. H.R.H. Princess Christian and other supporters of the Association were present. Miss Catherine Wood, who has been so intimately connected with the scheme from its commencement, has resigned her position as honorary secretary, which event was announced by Dr. Bezley Thorne. Dr. Bedford Fenwick and Dr. Bezley Thorne have succeeded Miss Catherine Wood, and are to be the honorary secretaries of the Association. A special badge was proposed and, later on, presented to Miss Kate Marsden in recognition of her investigations of the condition of the lepers in Siberia. Dr. Bezley Thorne read minutes of the last meeting, and a proposal that Miss Catherine Wood should be made a life-member of the Council was suggested. Certainly Miss Wood's constant labours on behalf of the Association, and her support of its interests throughout its entire existence, should entitle her to this or some similar compliment on her resignation. The short visit of the Misses Kenealy to Hamburg was kindly alluded to by H.R.H. Princess Christian, and the meeting closed with a vote of thanks to the chairman.

### The Training of Male Nurses.

INQUIRIES on this subject are frequently brought to our notice, but it seems impossible to get them satisfactorily answered.

The initial difficulty appears well nigh insuperable, for no general hospital, so far as we know, will undertake the training of male nurses, and until some institution for the sick is able to arrange for doing so, the very name is an anomaly. No one can claim the title of nurse, in the modern application of the word, without having earned the right to bear it by a full training in every branch of the profession.

To make a competent male nurse we must have a young man of good moral character and of industrious habits, with quiet manners, an even temper, and a sympathetic disposition; one whose tastes lie naturally in the direction of sick rooms, certainly not one who, having failed in many other callings, aspires to this as an easy mode of earning a livelihood with a minimum amount of personal trouble. An indolent man makes as incompetent a nurse as an unsympathetic one does. But granted the existence of a limited number of young men worthy to be trained, where are they to be taught? A supply of trustworthy and intelligent attendants for mental cases will be ensured as soon as all asylums for the insane give to their nurses complete theoretical and practical training during a course of at least two years, granting certificates for efficiency and good conduct at the end of the period. This, however, leaves us with no solution of the difficulties attendant on the other branches of the profession—surgical, medical, and fever-nursing.

The nursing in surgical male wards might be undertaken by men with very great advantage to the women whom they would set free for more suitable and congenial cases, and also it would be better for the doctors, students, and patients. The first step towards this is the instruction of suitable men, and if general hospitals have no space to accommodate the additional staff, and no wards to spare for the training of male probationers, the Poor Law Infirmaries could certainly furnish both.

Wherever there is an established trained Matron, it would surely be possible for the Medical Officer to arrange for one medical and one surgical male ward to be nursed by male probationers.

An experienced woman nurse for night, and one for day duty would at first be also necessary, and this supervision and instruction given by them, under the doctor, would leave no room for neglect or inadequate attention to patients.

Then, of course, as soon as a competent trained man were available for the post of charge nurse, he might be promoted to this responsible position. It would be obviously impossible to work a ward with a mixture of male and female probationers, but there should be no difficulty in the arrangement we suggest, although it would need discretion in formulating the plan, and choosing the persons to carry it out.

Of course there are a certain number of experienced male attendants already engaged in private work, and a few of these certainly merit the honourable title of nurse. We cannot say that the writer of the following letter, which appeared in a daily paper, gives evidence of either training or education, but it is signed a "Hospital Male Nurse": "Will you kindly allow me to say a few words specially for poor people to save them from an attack of cholera. It is very plain and not expensive, therefore I think it is my duty to let people know. Simply make a flannel bandage about three yards long and a quarter of a yard wide, and put that round the abdomen; keep it on day and night, and you can be sure that you will be more safe than if you took a lot of medicine, which sometimes does more harm than good. I can say that by experience." Poor man! Evidently he suffered from "too much of a good thing," whether his prescriptions were his own or those of qualified men. Yet "throw physic to the dogs" is hardly safe teaching from nurses who aspire to work "under the doctor's orders"! We saw a still more amusing letter from a person styling himself a male nurse the other day. He wrote of a sick person he had seen as "plainly suffering from pneumonia," but on the next page speaks of a doctor having been called in later on to see the case and "he said the patient had bronchitis." The diagnosis of the disease is the doctor's prerogative, and male nurses had better swiftly take this fact to heart.

It would be interesting to know whether a scheme for providing adequate training for suitable men would draw to the nursing ranks many educated candidates? We all know how attractive hospital life has become to refined women of the present day, but we imagine the suggestion that he should become a male nurse would offer small temptation to the would-be medical student. However, it is of more importance at present for us to try and find out two points—firstly, what kind of men are wishful for training? and, secondly, which institutions are likely and able to give the requisite instruction?

### Appointments.

[It is requested that successful candidates will send a copy of their applications and testimonials, with date of election, to THE EDITOR, The Lodge, Porchester Square, W.]

MISS MARY BARTLETT, M.B.N.A., has been appointed Sister in the Indian Nursing Service, and sails in H.M.S. "Serapis," on November 24th. She trained at Brighton Children's Hospital, Westminster Hospital, and Clapham Maternity, and has been engaged in private nursing for some years.

HULL SANATORIUM.—Miss King has been appointed Night Superintendent at this institution. Miss King was trained for three years at that excellent school, the Royal Hospital, Portsmouth, and has since done nine months' district nursing at Fareham.

YEOVIL HOSPITAL.—It is with pleasure that we learn that Miss Hyde has been appointed Matron of this hospital. She was trained at the London Temperance Hospital, and subsequently held the post of Sister there in the women's surgical wards. She was afterwards Matron at Dr. Walter's private hospital in Manchester.

GLASGOW SICK POOR AND PRIVATE NURSING ASSOCIATION.—Miss Berwick, from the Queen Victoria Jubilee Institute, Edinburgh, has been appointed as Assistant to the Superintendent of the Glasgow Sick Poor and Private Nursing Association. She was trained a year in the Edinburgh Children's Hospital, one year at the Incurable Home for Adult Training in Edinburgh, and two years and six months in monthly and district nursing in the Jubilee Institution.

### Notes and Queries.

### Queries.

(16) *Some Hints about Cannes.*—Can anyone tell me if in either Cannes or Monte Carlo there is work for a good masseur, are there any cheap hotels, and what is the average rate of living per week?—*Masseur.*

(17) "*Insanity and its Treatment.*"—Where can I get a copy of the book, "Insanity and its Treatment," by Dr. Blandford, new or second-hand?—*Mental Nurse.*

(18) *Free Training in Massage.*—Is there any hospital where a nurse, who cannot afford to pay, can obtain training in massage?—*J. T.*

### Answers.

(16) *Some Hints about Cannes* (*Masseur*).—Will some of our readers help us to answer this question? We should advise you applying to some of the nursing institutions down there. Unless you have introductions you will find work precarious on your own account. Miss Bryant, at San Remo, is starting a new home, and there are many others. Rooms are cheaper than any hotel.

(17) "*Insanity and its Treatment*" (*Mental Nurse*).—Ask Kimpton, 82, High Holborn, if he has a second-hand copy. We are not sure of the publisher, but will find out; perhaps Mr. Kimpton could tell you.

(18) *Free Training in Massage* (*J. T.*).—We do not know of one. Do you wish to become a regular masseuse? If you will write to "Nursing" more fully, perhaps we could suggest some plan to you.

### Some Australian Experiences.

(*Continued from page* clxvii., *Vol. XII.*)

My next hospital experiences were not quite so pleasant, although they brought me in contact with such charming people. I shall never regret leaving lovely old Sydney for "fresh fields and pastures new," but look forward with much pleasure to my return to the most delightful (in my eyes) of all the Colonies—Queensland.

The "Little Bay" Hospital at Sydney, N.S.Wales, is kept for fever cases exclusively, and is situated in a wonderfully pretty, although rather wild neighbourhood, facing the ocean. "Little Bay" is next to the famous Botany Bay, which still remains much as it was when discovered by Captain Cook over a hundred years ago. Although so many people seem to think it is a settlement, no actual township exists, Botany proper being some distance from the Bay.

The Coast Hospital, as it is more generally called, is arranged in separate buildings, similar to our London Fever Hospital, Haverstock Hill; there are the typhoid wards, scarlatina wards, measles wards, and at a distant part of the place, the leprosy tents. These latter are in charge of Chinamen, as Europeans seldom suffer from this fearfully loathsome disease.

Although the hospital is over seven miles from Sydney proper, there is telegraphic communication between it and the city, and calling at the Health Office in the morning anyone may hear how their friends are going on. The whole place is most comfortable, the wards are nice and cool, and sea breezes give that indescribable freshness to the air, while a very efficient nursing staff and resident medical officer look to the patients' wants.

There is a convalescent ward, to which many patients from the "Prince Alfred" and "Sydney Infirmary" are sent. Many scarlatina cases go there during the desquamation period, to make more room in the City Hospital.

The great objection to it is its distance from Sydney, the journey is so trying for typhoid cases, although there is a most comfortable ambulance, always accompanied by a trained nurse, so that the journey is made as comfortable as possible for the patient.

I took a scarlatina case down once, and was most pleased with the whole place; the Doctor took me all round. At the time I speak of the train only went as far as Botany, leaving three miles to walk to the hospital. Now a bus runs between the two on visiting days, so on the whole a visit to the Coast Hospital is a pleasure.

The Children's Hospital at Glebe Point is not quite such a success, but the great popularity of the "Prince Alfred" accounts for this to a large extent, more acute cases are treated there, and the "chronic" ones seem to get up to the "Glebe." There are several first-class "Private Nursing Homes" in Sydney—a private speculation of the doctors who have qualified Sisters in charge, and nurses according to the number of patients.

The accommodation for sickness in the private houses generally, is so limited, that these "Homes" are a great comfort, more especially for people from the country, and the way in which they are patronised proves they are appreciated.

Of the inner working of the Sydney Infirmary, I know very little. It is an old wooden building, a fault they are trying to remedy by putting up a stone one, but want of funds is rather an impediment to progress.

A very beautiful Convalescent Home has recently been opened at Cambden, one of the loveliest districts in N. S. W. It was known as the "Meadow lands" in the old days of the colony, and was at that time famous for wheat growing, but the "rust" appeared, and did so much damage, it had to be discontinued.

The Hon. Jas. White has his training paddocks and stables quite near, and round about the district are "country seats" equal to many in England, such as "Wivenhoe," "Oran Park," "Oxly Manor," &c., and "Cambden" town is as curious a little place as you could wish to see.

The roads round about are splendid, and the scenery not unlike the west of England.

I am not sure whether Mr. Paling is the founder of this "Home"; at any rate, I am safe in saying he contributed largely to its foundation. It is doubtless a great boon, situated as it is, only forty miles from Sydney; it is easily reached by train t Campbelltown, and then the steam tram runs to "Cambden."

There is a smaller "Home" at Moss Vale, about fifty miles from Sydney, so you see they are "up to date" with their institutions.

There are no workhouses in the colonies, but homes for incurables and aged men and women. One is at Parramatta, and there is a very large one at Liverpool Plains; they are called "benevolent institutions," and very comfortable and well cared for they look. I remember one of the cases I saw at one of these places was that of an old man who was under the delusion that he was a woman, so, to please him, they allowed him to wear female attire. He had a cotton gown, apron, and large white sun-bonnet on, and he was dusting away quite happily. I was told he was a gentleman by birth, and had been educated at Oxford.

One more hospital in Sydney I must mention, and that is "St. Vincent's," Darlinghurst, managed and nursed by the Roman Catholic Sisters. It is a beautiful place, and so well kept; people of any creed are nursed there.

I think you will agree that for a young colony, like New South Wales, these institutions are a great credit. Up country from Sydney the institutions are also most comfortable, and nearly all are in charge of trained nurses. There is no doubt that nursing by skilled people is most thoroughly appreciated there.

After fifteen months I left the "Prince Alfred," and set off on my travels again, being most anxious to see as much of the country as I could. I gladly went with a patient to the lovely Blue Mountains, about sixty miles from Sydney. We made Mount Victoria our headquarters, then took drives or rambles round about, and we also visited the Lithgo Valley, a coal mining district. All about Mount Victoria the scenery is certainly most charming, and the country is covered with beautiful flowers and foliage of all descriptions.

The beautiful blue haze which envelops the mountains sheds a softness over everything, and the view from Gouett's Leap (a high, precipitous point, from which, it is said, an escaped convict jumped rather than be retaken) is most grand. It is like looking across a vast sea of bush and scrub.

After my return I again went "up country" with a lady to another part of the colony, and had a most delightful time, plenty of riding, driving, and rambles through the bush.

My experience of country or bush life was a most pleasant one at this time, and I felt the benefit of the change also.

My chief companions were the dogs, and when out on the tramp we often started a hare, or discovered an opossum in a hollow log; then there would be intense excitement. Being the only Englishwoman on the place, I was very proud when one day I brought home a fine hare we successfully "coursed," although I confess that I got what the boys call "rather pumped."

I returned to Sydney, and, after a short stay, went over to Melbourne. I went by train to see the country, which is not very remarkable for beauty; in fact, I was greatly disappointed. We stayed a little at Wagga Wagga, where the famous Tichborne claimant came from, and afterwards we crossed the border and passed through the country where the notorious Kelly gang of bushrangers carried on such high jinks, and we also saw where they were taken.

(*To be continued.*)

### Everybody's Opinion.

*[Correspondence on all subjects is invited, but we cannot in any way be responsible for the opinions expressed by our correspondents. No communications can be entertained if the name and address of the correspondent is not given, or unless one side of the paper only be written on.]*

### A PLEASANT LECTURE.

"Nurse M. C." writes from the British Lying-in Hospital: May I tell my fellow "students in midwifery" of the pleasant evening we spent last night at the Midwives' Institute, 12, Buckingham Street, Strand. We received invitations—through our Matron—from Mrs. Nichol, the hon. secretary, to attend a lecture on "Midwifery." Several of us went, as much from a sense of duty as anything else. We were little prepared for the bright and instructive lecture that followed; I for one came home so refreshed, that instead of complicated cases chasing each other through my brain, I slept the sleep of what is supposed to be the just, and this morning I feel I need no longer feel unhappy over anything, because Dr. Leith's descriptions were so distinct and understandable. Also, Dr. Leith was very kind and liberal in his views of midwives, "who are to be controlled by and bye," and we all felt thankful that we had at least one true friend and well-wisher amongst those who are sometimes such stern judges of our abilities. We are all desiring another invitation, and if Dr. Leith will give us a little more of his valuable time, we shall be most grateful, and Mrs. Nichol makes all midwives so welcome.

### Presentations.

Miss Jessie Brown, Assistant Matron at the Huddersfield Nurses' Home, was, on Thursday, the 13th inst., presented by the staff with a travelling clock as a parting present on the occasion of her leaving England on the 19th to go to relatives at Kylertown, Pa.

Devon and Exeter Hospital.—On October 19th, Miss Millar, Superintendent of Nurses at the Devon and Exeter Hospital, was presented with a handsome set of silver backed brushes, bearing her monogram, as a token of the esteem in which she is held by her nurses. Miss Millar is leaving for Cairo, and takes with her the good wishes of the nursing staff.

Paignton Cottage Hospital.—Miss Cheatle, Matron of the Paignton Cottage Hospital, has been presented by the patients and staff with a very handsome writing-desk and inkstand in recognition of her kindness and valuable services. Miss Cheatle was appreciated by all those with whom she worked, and the poor of Paignton have lost a good friend.

Devon and Exeter Hospital.—On the 24th inst. Nurse Florence Hayes was presented with a beautifully embroidered screen by the Matron and Nurses of this Institution. Nurse Hayes has been at this hospital three years as Probationer and Charge nurse, and leaves with the good wishes of her fellow nurses.

### The Dundee Nurses.

On Friday, the 21st, H.R.H. the Princess Louise and the Marquis of Lorne visited Dundee. They had an enthusiastic reception, and went to see the Nurses' Home working in Dundee in connection with the Q.V.J.I.N. The Princess Louise is the President of the Scottish Branch of the Jubilee Association, and is nobly using her influence to promote the work in every direction.

### THE SYMPATHY OF CHRIST.

When we are sick and weak we are often so taken up with our pain that we are seldom in a frame of mind to read and study the word of God. Some may be so weak indeed and so racked with pain that even though they are the Lord's people they cannot in such circumstances recall any comforting passages of God's word. But let us try to remember how blessed it is that we have a High Priest Who is touched with the feeling of our infirmities, and Who is continually bearing our names before His Father and our Father. Jesus sympathises with us, and through that sympathy we obtain mercy, and find grace to help in time of need. He is continually making intercession for us, and is able to save to the uttermost. Yes, for our blessed Lord has been a man Himself, has trod the path of sorrow, and has been tried in every way. He knew what it was to be weary, to suffer hunger and thirst, and to groan in spirit and to weep.

Moreover, we are tempted and enticed because we have sin in us. He was likewise tempted, although without sin. He can, therefore, doubly sympathise with us, "for in that He Himself hath suffered; being tempted He is able to succour them that are tempted."

"He in the days of feeble flesh
Poured out His cries and tears,
And in His measure feels afresh
What every member bears."

We, therefore, do not go to any earthly friends for help, but to the great High Priest of our profession. What a wondrous place to get consolation, help, and sympathy from the very throne of God Himself, the God of all comfort! Therefore, when we are called to walk through the valley of the shadow of death itself, we will fear no evil, for He will be with us; His rod and His staff will comfort us.

### Wants and Workers.

The Matron of the Miners' Hospital, Redruth, Cornwall, would gratefully receive an old piano for the use of the convalescent patients, many of whom are musical; in the long winter evenings a piano would be a great boon.

### The Result of a Chill.

### IV.—A SATISFACTORY CONCLUSION.

That night he re-read Sybil's letter of thanks. Its tone of gratitude was pathetic. He discerned plainly that she had been in straits and serious despondency, and that the good news was almost overwhelming. She spoke of coming to town to see her picture in its place of honour, framed and numbered, and hanging amidst the productions of acknowledged artists. It was very agreeable to a man of his sympathetic mould to reflect that he had plucked an ambitious mortal from the torture of frustrated hopes.

When Sybil arrived in London she went direct to Regent's Park. She was strongly impelled to visit the Gallery on her way, but first she considered it a duty to thank Mr. Lancaster in person. Her life had fostered a kind of shyness, and it was with a slightly fluttering heart that she asked for Cornwallis Avenue at the Park Gates. Mr. Lancaster was within, and a servant preceding her with a visiting card, showed her into a nondescript and well-furnished apartment, that only needed feminine hands to transform it to a drawing-room. One of the first objects that met her eye was her water-colour sketch standing on a table. Her face flushed at the sight of it. There it was, with all its shortcomings, in a conspicuous position in the room of a leading art critic. She sat musing happily on the turn in her fortune till Arthur Lancaster entered, and shook her hand warmly. His face was thinner, she thought, but the sunburn had not quite faded from it. In a few moments she was at her ease, and talking of her future prospects.

"You must not think of abandoning painting," said the critic. "The root of the art is in you, for the buds have already shown themselves. But remember an uphill life is before you."

"I am not afraid of hard work," she said. "You have greatly cheered me—more than I can express. I have come to thank you."

"I deserve no thanks," he returned. "I am glad you have come, though. We will go together to the Arcade Gallery, and I will show you where your picture is strong, and where it is weak. But let me supplement the apology I made in my letter. I ought to have told you my name when I was at Hollacombe. Do you forgive me? I couldn't withstand the temptation of concealing it when you quoted me. Were you not surprised to find that I had played this little trick upon you, Miss Ruthven?"

"At first I could scarcely believe my eyes," she answered.

"Tell me, who or what did you think I was?" he said, laughing.

"I thought you an artist."

"Well, perhaps that was natural. I surmised as much."

While they went through the wide West-End thoroughfares in the noon sunlight, Sybil's spirits rose to a point of ecstasy. All the depressing fears of the past month were dispelled and forgotten, and her heart glowed with gratitude to the man who had aided her to self-confidence and hope. In the glad excitement of the moment when they stood before the picture, her eyes were humid, and her voice faltered when she said,

"I cannot think that you have bought that poor picture on its merits only. Was it not to encourage me?"

He looked down at her.

"I bought it on its merits," he said.

"But I fear it has none."

"That is not flattering to my discrimination."

"No, I don't mean that. Oh, dear! I mean it is crude."

"Granted. Still, it is honest, and it gives me more pleasure than any other picture in this gallery."

"I am glad to win a friend who has already shown me such kindness."

"Please don't talk of kindness again. True friends don't reiterate thanks for small favours. I wish to be a friend to you. You have asked for my counsel, which implies that you repose a certain amount of confidence in my judgment. Let me advise you, then, to leave the wilderness till the summer, and get a well-fitted studio in town. You must not remain in obscurity if you wish to make a name."

"But I love the country, and I hate London life!"

"May be; so do I; but will you act upon a friend's advice?"

"Willingly, if you think it for the best. But how can I expect to earn enough to rent a studio? Do you think that I can sell my pictures?"

"I can find a market."

"Yes, but really on their merits?"

"Yes, on their merits. But why do you harp on that single note?"

"Because now I long to go on. You have made me aspire."

"It is fatal to aspire—too much. So long as you stay in the wilds you will aspire, and do little else. I am giving you sound business advice. You must 'work up a connection,' as the tradespeople say. I can find you a studio for very little money. Now will you be practical, Miss Ruthven?"

"I will do what you advise," she answered.

A few weeks after Sybil and Hilda Ruthven were sharing a studio in Cornwallis Avenue. It was on the opposite side of the way to Mr. Lancaster's flat; and while the critic sat at his desk he could see the girls at their easels. One day, towards the end of May, Arthur Lancaster put down his pen, lit a cigarette, and stood with his hands in his pockets looking across at the studio. Hilda was out shopping, and Sybil was sitting by the open window.

"She is thinking about Hollacombe," mused the critic. "I know she longs to get away from town this fine weather. I wish I could wean her from her passion for Devonshire."

Suddenly he threw the cigarette away, got his hat, and went across the road. As he was a persistent caller Sybil was not surprised to see him enter the studio, and sit down on the opposite side of the window. But she was unable to ascribe a reason for his gloom that afternoon.

"Isn't this weather glorious?" she said. "Oh! how I long for Hollacombe."

"I thought so," he returned, "I know you wish to be flitting, and I am morose, almost savage, at the thought of your going. Selfish, isn't it?"

"It is a kind of selfishness one ought to find it easy to condone," she said. "But how can you be wretched at the prospect of a visit to Italy? It seems too absurd."

"Had I a congenial companion—yourself, for example—I should be anything but gloomy. Sybil, I am not going to wait any longer. You shan't run away into the wilderness and leave me to mope here till autumn, without knowing what separation from you means to me. I shall be desperately miserable."

"I wouldn't willingly cause you misery. Shall I stay?"

"Yes, till autumn, say," he answered.

"Why till autumn?"

"Because—well, because then, if you will only marry me, Sybil, we can both go to Italy, or Hollacombe, or anywhere else you choose."

And before he left the studio she chose to go to Italy.

Geoffrey Mortimer.

## Figures and Tables

**Figure f1:**